# Differences in study workload stress and its associated factors between transfer students and freshmen entrants in an Asian higher education context

**DOI:** 10.1371/journal.pone.0233022

**Published:** 2020-05-15

**Authors:** Kin Cheung, Tsz Leung Yip, C. L. Johnny Wan, Hilda Tsang, Lillian Weiwei Zhang, Anna Parpala

**Affiliations:** 1 School of Nursing, The Hong Kong Polytechnic University, Hong Kong, China; 2 Department of Logistics and Maritime Studies, The Hong Kong Polytechnic University, Hong Kong, China; 3 Centre for University Teaching and Learning (HYPE), Faculty of Educational Sciences, University of Helsinki, Helsinki, Finland; University of Macau, MACAO

## Abstract

Unlike the studies of freshmen entrants, the learning experiences of community college transfer (CCT) students in the receiving university is a topic that has only started to gain attention in recent decades. Little is known about the differences between CCT and freshmen entrants with regard to their study workload stress and its relationship with their perceptions of the teaching and learning environment, approaches to learning, self-efficacy and generic skills. The purpose of our study was to address this gap. This was a cross-sectional survey study conducted from April 2018 to November 2018 in a university in Hong Kong. The HowULearn questionnaire was adapted to local usage and validated for data collection. In total, 841 CCT students and 978 freshmen entrants completed the survey. The respondents were aged between 19 and 52 years (mean = 21.6, SD = 1.92), and 66.0% were women. The HowULearn questionnaire was determined by factor analyses to have eight factors. The reliabilities of the eight factors were found to be acceptable (Cronbach alphas = 0.709–0.918). The CCT students scored significantly higher than the freshmen entrants for perceived study workload stress and surface approaches to learning, but lower on teaching for understanding & encouraging learning, peer support, and self-efficacy beliefs. The surface approach to learning, deep & organized studying, alignment & constructive feedback, and generic skills were found to be predictors of study workload stress in both groups of students, and in the overall student data. This study has shown that CCT students and freshmen entrants differed with regard to their study workload stress and learning experiences. Our findings provide a message, both for educators in higher education and policy makers in the government—there is not a one-size-fits-all approach to different student populations when it comes to enhancing their learning experiences.

## 1. Introduction

In addition to direct admission to university after graduation from secondary school, the higher education systems in most western and eastern countries provide an alternative pathway towards an undergraduate degree [1]. For various reasons, graduates from secondary schools might gain admission to community colleges to obtain associate degrees or higher diplomas [2]. After graduating from the community colleges, they can be accepted into the third year of their chosen four-year degree programmes, based on a credit unit transfer system [1]. In the literature, these students are called either vertical transfer students [3] or community college transfer (CCT) students [4]. In this paper, we refer to them as CCT students. Nowadays, the admission of CCT students is increasingly common in universities [5] worldwide. Despite their growing presence, CCT students’ learning experiences when they enter university have not been taken into consideration thoroughly [5], particularly in Asian or European countries. Most research on CCT students has been conducted in the United States (US) [4,6]. Study-related stress is well known as detrimental to university students’ physical and psychological health [7], as well as academic performance [8,9]. However, there is a lack of research studying the factors associated with study workload stress in CCT students and how these compare to the experiences of students who enter university directly from secondary school to undertake 4-year programs, who are referred to as freshmen entrants [4]. In order to support both student groups, there is an urgent and important call to study their similarities and differences.

### 1.1. Challenges faced by CCT and freshmen entrants and associated with their study workload stress

Both CCT and freshmen entrants experience a certain level of uncertainty in their process of transition to the university learning environment. The limited studies comparing the two group of students have reported that CCT students experienced more psychological issues than freshmen entrants [2,10], with heavier study workloads [2,8,11,12]. It has been found that the stress induced by heavy study workloads could affect psychological health [13]. In addition, the issue of “*transfer shock*” (i.e., a drop in GPA immediately after transferring to university study) for CCT students has been discussed well in the literature [6,14,15]. Even though the results of studies are inconsistent, CCT students, in general, have higher attrition rates [4,16,17] and lower academic performances than freshmen entrants [4,18,19]. Flaga [14] argued that academic performance is a consequence of a complex set of processes. She also proposed that the negative impacts (such as poor academic performance, and perhaps study workload stress) could be related to the CCT students’ transition to the new learning environment.

In addition to transfer shock, these students also experience campus culture shock [6] because of the various differences between community colleges and universities. From the academic perspective, CCT students have described the teaching and learning approaches in university as different from those in the community college. For instance, they found the teaching pace to be faster and the assessment focused more on writing [20]. Furthermore, the students also needed to establish new peer-support networks [21,22]. The teaching and learning environment has been found to have a significant association with academic performance [8,9], while academic frustration can lead to dropping out from the study [23]. However, university faculties and administrators usually assume that the differences between community colleges and universities are minimal and not detrimental [6]. On the contrary, the issues relating to transition from secondary school to university are more visible. For instance, Kantanis [24] reported that secondary school leavers have social transition issues because many of them are also transiting from adolescence into adulthood and they need to develop new networks in a new learning environment. In addition, freshmen entrants make up the majority of the undergraduate student population. In the US, for example, 75.1% of the degree earners in 2016/17 were freshmen entrants with no prior awards and 20.3% were associate degree holders [25]. Thus, research [26,27] and resources [28] devoted to freshmen entrants are more prominent. This inequality might contribute to CCT students and freshmen entrants having different learning experiences. Furthermore, with inadequate transfer of the credits earned from community college to university studies, as mentioned, CCT students experience heavy study workloads and study-related stress [2,8,11,12]. Another possible difference is that CCT students and freshmen entrants might have different academic-related experiences in university [29]. CCT students often have expectations about “class experiences, workload distinctions, assessment models and interactions with faculties” (p.171) [29] that do not match their actual experiences. Thus, CCT students might have higher study workload stress than their counterparts.

Previous research has examined the differences between CCT students and freshmen entrants in academic achievement (i.e., GPA), and retention rates [21]. As discussed, CCT and freshmen entrants might have different learning experiences in university due to their different routes of entry. Furthermore, study workload stress and its associated factors, such as approaches to learning, experiences of the teaching-learning environment, self-efficacy, and generic skills, are all important in the light of academic performance [30–34]. However, comparisons between the CCT students and freshmen entrants in these important factors have not yet been examined. In the next section, these factors are presented in more detail.

### 1.2. Approaches to learning

Approaches to learning describe students’ intentions and study processes [35–37]. Three approaches to learning can usually be identified: deep approaches, surface approaches and organised studying. Students who apply deep approaches to learning aim at understanding and concentrate on analysing and relating ideas, whereas those who apply surface approaches concentrate on memorising information, resulting in fragmented knowledge bases [35,36]. Recently, the term “unreflective approach” has been suggested to describe the surface approach because students who adopt this are unreflective, struggling to relate ideas and focus on memorisation [38]. The third approach, organised studying, includes good time-management skills, self-regulation and effort in studying and refers to how systematic students are [39]. It also relates to a sense of responsibility in studying [40]. Previous research on students’ approaches to learning has found that there are differences the learning approaches of eastern and western students. Sakurai and colleagues [41] and Zhu and colleagues [42] indicated that eastern students are better adapted to surface approaches than western students. Some studies in Japan and China have found that their students employ both surface and deep approaches to learning simultaneously or in series [30–34,43]. Furthermore, students’ use of organised studying is still rather unexplored in the Asian context.

### 1.3. Teaching-learning environment, self-efficacy and generic skills

Students’ approaches to learning have been found to be related to their experiences of the teaching-learning environment [44–49]; their self-efficacy beliefs [50–52]—based on context-specific assessments of one’s own ability to perform a task successfully [53]; generic skills such as problem solving; and their perceptions of study workload [54]. Concerning the self-efficacy beliefs, Prat-Sala and Redford [52] reported that academic self-efficacy beliefs were linked positively with deep approaches to learning but negatively with surface approaches. Other previous research has shown that perceived study workload is associated positively with surface approaches [36,54–60]. Finally, there is evidence that deep approaches to learning are related to better learning outcomes than surface approaches, such as memorising and struggling with a fragmented knowledge base [61,62]. Another factor found to have contributed to better academic achievement is exposure to good teaching, clear goals and appropriate assessment [63]. A similar result was found by Diseth and colleagues [64], that the experiences of good teaching and appropriate assessment were related to academic achievement.

The existing literature has investigated the differences between CCT students and freshmen entrants in terms of their academic achievement. There is a need to explore the differences in their perceptions of the teaching and learning environment, approaches to learning, self-efficacy and study-related workload stress. These new insights can help to explain the variations in academic achievement. To address this need, the first aim of this study was to test and validate the instrument called HowULearn (Prev. Learn [65]) in an Asian context. HowULearn has been shown to be a robust instrument in a European context, but it has also been used as a quality tool in different institutions to enhance teaching and learning. The institutions have become more aware of what constitutes quality teaching and learning [61,62,66]. Moreover, there has been interest in the content of each scale measuring academic quality and, therefore, the focus has been on item level as well [67]. The items provide concrete ideas for enhancing quality.

Second, the differences between CCT students and freshmen entrants in their study workload stress, perceptions of the teaching-learning environment, approaches to learning, generic skills, and self-efficacy were examined. Last, the factors associated with study workload stress were investigated. Our study had three hypotheses: (1) the HowULearn instrument would be applicable to an Asian educational context; (2) compared with freshmen entrants, CCT students would have higher study workload-related stress, perceive less desirable on their teaching-learning environment, approaches to learning, generic skills and self-efficacy; and (3) the factors associated with study workload stress would be different for the two groups.

## 2. Methods

### 2.1. Design and participants

This was a cross-sectional survey questionnaire study using convenience sampling. All full-time government-funded undergraduate students from a university in Hong Kong were invited to participate in the study. Invitations were issued via email, posters and in-class promotion. The students were invited to fill in an online questionnaire between April and November 2018. Local undergraduates who had been admitted directly from secondary school and those who had come from community college studies were included, but international students were excluded. Ethical approval (HSEARS20180104005-01) was obtained from the Human Subjects Ethics Sub-committee of the Hong Kong Polytechnic University.

In Hong Kong, a quota is assigned to government-funded universities to accommodate CCT students to complete the four-year university undergraduate studies in two years. These two-year CCT students are referred to as 2yCCT students. The freshmen entrants who participated in this study were on a path to complete their undergraduate studies in four years. A small number of these local freshmen entrants might have completed one-year or two-year community college or university study either in Hong Kong or overseas, but were considered as freshmen entrants because they had the same study duration and support resources as those admitted straight from secondary school.

### 2.2. Instrument

The online questionnaire (see supplementary information) started with the study information sheet and implied consent. The respondents were asked to indicate if they were 2yCCT students. Personal information, such as year of birth and gender, was collected. This was followed by five sections from the HowULearn questionnaire (previously named “Learn Questionnaire”, focusing on (1) student experiences of the teaching-learning environment; (2) approaches to learning; (3) generic skills; (4) self-efficacy beliefs; and (5) study workload stress [65].

The section in HowULearn measuring students’ experiences of the teaching-learning environment originated from the Experiences of Teaching and Learning Questionnaire (ETLQ) [68]. The HowULearn Questionnaire has been developed further from the ETLQ over many years based on extensive statistical analysis as well as student and expert interviews in many different contexts [65,67,69–71]. It is based on a literature review and an analysis of existing inventories measuring students’ experiences of teaching-learning environments or academic quality [68,69]. All the scales that were used in the study are based on theories of good teaching; more precisely, they measured dimensions that support students’ deep approaches to learning. Their origins were in curriculum development, which suggests ideas of how curriculum can help students to develop their understanding, for example, by bringing teaching and assessment methods into line with each other [72]. Some of the scales were drawn from the theory of how teaching methods can provoke students’ interest and help them to enhance learning [73,74], and some of them emphasised the balance of teachers’ and students’ roles in supporting learning [75]. A 5-point Likert scale (1 = totally disagree, 5 = totally agree) was used to measure teaching-learning environment experiences. In the Finnish data, six factors emerged from the 22 items measuring different aspects of quality teaching: (1) teaching for understanding, (2) alignment, (3) staff enthusiasm and support, (4) interest and relevance, (5) constructive feedback, and (6) support from other students [69].

The HowULearn instrument had 12 items measuring approaches to learning [65]. A 12-item version in the HowULearn was modified from the Approaches to Learning and Studying Inventory (ALSI) [39] and the Learning and Teaching questionnaire (LSQ) [68]. In addition, two items were from the Revised Learning Process Questionnaire (R-LPQ9) [31]. In these items, students were asked to describe how they had been studying in general. A 5-point Likert scale (1 = totally disagree, 5 = totally agree) was used. The items measuring approaches to learning consisted of three scales, Deep approach, Surface approach and Organised studying. The HowULearn-questionnaire and the scales for approaches of learning are used widely and have been validated in Finnish and international contexts (e.g. [46,65–67,71,76–78]).

Generic skills were measured by seven items from the HowULearn questionnaire [65]. The students were asked to evaluate how their university studies had developed different generic skills such as analysing and structuring information, critical thinking, applying knowledge, collaboration and communication skills and developing new ideas. The items were derived partly from the review of the literature and partly from examinations of previous inventories, for example, Course Experience Questionnaire (CEQ) [79,80]. A 5-point Likert scale (1 = totally disagree, 5 = totally agree) was used.

To measure the students’ self-efficacy, a scale was constructed based on the Motivated Strategies for Learning Questionnaire [81]. Five 5-point Likert-scale items were modified to suit the academic discipline level of analysis rather than the course level [65]. Self-efficacy refers to students’ self-appraisal of their ability to master academic tasks, which includes their judgements about their ability to accomplish a task as well as their confidence in their skill to perform that task. Perceived study workload stress was measured using three items from the HowULearn questionnaire [65] using a 5-point Likert scale (1 = totally disagree, 5 = totally agree).

For this study, the questionnaire was reviewed by a panel of eight local experts in the education field, and modifications were made to fit the local use. The modified questionnaire was then reviewed by a panel of nine overseas and local experts in the education field to determine the content validity index (CVI). A CVI of 0.99 was found, which was higher than the acceptable level of 0.75 [82]. Eleven undergraduate students were invited to fill in the questionnaire to test its readability and appropriateness. Minor changes were made to some words.

### 2.3. Data analysis

SPSS analytical software version 25 was used for the data analysis. We conducted exploratory factor analyses (EFA) for each construct by using the general rule of an eigenvalue > 1 [83], and used the maximum likelihood extraction method and oblimin rotation. The Kaiser-Meyer-Olkin (KMO) test was conducted to measure the sampling adequacy. Cronbach’s alpha statistics were computed to test the scales’ internal consistency. The presence of multicollinearity among the independent variables was examined by the tolerance values and the variance inflation factor (VIF) for the data included in the analysis. Confirmatory factor analyses (CFA) using SPSS AMOS 25 were conducted on the original study factors as well as the new factors that emerged from EFA. The fit of the model was assessed using the chi-square test of model fit, the goodness-of-fit index (GFI), the Tucker-Lewis index (TLI), the comparative fit index (CFI), and the root mean square error of approximation (RMSEA). There were not many missing data, as mandatory answers had been set. Items with missing data were excluded from the factor analysis.

The average scores for each factor were computed for each participant. The differences between the scores of the 2yCCT students and freshmen entrants on the scales and individual items measuring students’ perceptions of the teaching-learning environment, approaches to learning, self-efficacy, generic skills & development, and study workload & stress were analysed using a T-test for independent samples.

Pearson’s correlation test was used to test the correlations between the scales of the teaching-learning environment, approaches to learning, self-efficacy, generic skills & development, and study workload & stress. Variables with statistically significant correlations with study workload and stress were selected for the linear regression analysis (forward) to explore the strongest predictor of study workload and stress.

## 3. Results

### 3.1. Characteristics of the students

Of the 1,819 participants, 841 (46.2%) 2yCCT students and 978 freshmen entrants (53.8%) responded and completed the survey. The students were from 28 departments involving all faculties and schools. The sample consisted of 34.0% men and 66.0% women, aged 19 to 52 years (mean = 21.6, SD = 1.92). The gender ratio and mean age of our study students were consistent with those in the university records. Most of the participants were in the first year (40.0%) or second year of study (32.0%) and the rest were in the third (18.0%), fourth (9.0%) or fifth years (1.0%) (Table 1).

**Table 1:** Characteristics of 2yCCT students (n = 841) and freshmen entrants (n = 978).

Characteristics	2yCCT Students	Freshmen Entrants	Overall	University Records
Gender	N = 841	N = 978	N = 1819	N = 14,799
Female	541 (64.3%)	659 (67.4%)	1200 (66.0%)	7626 (51.5%)
Male	300 (35.7%)	319 (32.6%)	619 (34.0%)	7173 (48.5%)
Age	N = 824	N = 962	N = 1786	N = 14,799
Mean (SD)	22.26 (1.77)	21.09 (1.89)	21.63 (1.92)	21.58 (1.79)
Range	(20–52)	(19–43)	(19–52)	(17–52)

### 3.2. Factor analyses

The factor structure of the items measuring the students’ experiences of the teaching and learning environment showed more variations than in previous studies [69]. (Tables 2–5) shows the factor analysis of the 6 factors and their attributes (items). These 6 factors can be viewed under the revised model proposed by Parpala and colleagues [69]. The revised model offers a systematic framework that enables the comparisons and contrasts between the 2yCCT students and freshmen entrants. Only three factors emerged that were labelled as “*Teaching for understanding & encouraging learning*”, “*Peer support*”, and “*Alignment &* c*onstructive feedback*” (Table 2), and accounted for more than 50.0% of the total variance. These were different from the original study with six factors. The same observations were found for approaches to learning and studying. Only two factors were loaded and labelled as “*Deep approach and organized studying*” and “*Surface approach*” (Table 3), which was different from the original study with three factors. They accounted for about 40.0% of the total variance. Only one factor was loaded and accounted for generic skills and development (Table 4), which was different from the original study with four factors. It accounted for more than 45.00% of the total variance. One factor was loaded for self-efficacy, and study workload stress (Table 5), which was similar to the original study.

**Table 2:** Comparison between 2yCCT students and freshmen entrants on the factor analysis in the subscale of teaching and learning environment.

Teaching and Learning Environment		2yCCT Students n = 839, KMO = .965			Freshmen Entrants n = 978, KMO = .964			Overall N = 1817, KMO = .968		
Questions		Factor Loading	Variance 55.82	α	Factor Loading	Variance 50.32	α	Factor Loading	Variance 52.91	α
Teaching for understanding & encouraging learning (11 items)		8 items	3.84	.911	12 items	44.13	.910	11 items	46.67	.918
1	It is clear to me what I am expected to learn in subjects	-.665			.611			.679		
2	We are allowed some choices over what aspects of the subject to concentrate on in subjects	-.520			.305			.448		
3	What we are taught seems to match what we are supposed to learn	-.813			.792			.826		
4	I can see the relevance of most of what we are taught	-.848			.780			.834		
5	Subjects have given me a sense of what goes on "behind the scenes" in the subject area	-.714			.654			.700		
6	The teaching helps me to think about the evidence underpinning different views	-.512			.678			.615		
7	Teaching encourages me to relate what I learned to issues in a wider context	-.477			.705			.602		
9	I found most of what I learned in subjects really interesting	-.515			.577			.597		
		8 items	49.28	.891						
10	Academic staff try to share their enthusiasm about the subject with us	.619			.500			.372		
12	Academic staff are patient in explaining things which seem difficult to grasp (deleted)	.650			.377			.257		
13	I enjoyed participating in subjects	.408			.535			.509		
14	Academic staff help us to see how we are supposed to think and reach conclusions in subjects	.641			.484			.347		
Peer support (3 items)					3 items	2.89	.767	3 items	2.96	.771
8	Students support each other and try to give help when it is needed	.595			.709			.684		
11	Talking with other students helps me to develop my understanding	.634			.710			.723		
15	I can generally work comfortably with other students	.666			.723			.727		
Alignment and constructive feedback (7 items)					7 items	3.29	.882	7 items	3.28	.889
16	The subjects provide plenty of opportunities for me to discuss important ideas and topics	.497			-.305			.330		
		6 items	2.71	.888						
17	I receive enough feedback about my learning (e.g. assignment work)	-.576			-.751			.723		
18	It is clear to me what is expected in the assessed work (e.g. final examination)	-.496			-.540			.528		
19	I can see how the subject assessment fits in with what I am supposed to learn	-.638			-.420			.516		
20	The feedback given on my work helps me to improve my ways of learning & studying	-.825			-.570			.749		
21	The subject assessment helps me to make connections to my existing knowledge or experience	-.650			-.384			.546		
22	The feedback given on my subject assessments helps to clarify things I hadn’t fully understood	-.786			-.568			.700		

**Table 3:** Comparison between 2yCCT students and freshmen entrants on the factor analysis in the subscale of approaches to learning and studying.

Approaches to Learning and Studying		2yCCT Students n = 841, KMO = .845			Freshmen Entrants n = 978, KMO = .844			Overall N = 1819, KMO = .847		
Questions		Factor Loading	Variance 39.92	α	Factor Loading	Variance 40.05	α	Factor Loading	Variance 39.89	α
Deep approach and organized studying (8 items)		8 items	28.12	.837	8 items	28.03	.837	8 items	28.00	.837
2	I put a lot of effort into my studying	.479			.512			.498		
4	On the whole, I’ve been systematic and organized in my studying	.723			.657			.682		
5	Ideas I’ve come across in my academic reading set me off on long chains of thought	.601			.474			.527		
6	I look at evidence carefully to reach my own conclusion about what I’m studying	.704			.588			.641		
8	I organize my study time carefully to make the best use of it	.632			.678			.657		
10	I carefully prioritise my time to make sure I can fit everything in	.598			.729			.671		
11	I try to relate new material, as I am reading it, to what I already know on that topic	.634			.693			.669		
12	I try to relate what I have learned in one subject to what I learn in other subjects	.636			.654			.653		
Surface approach (4 items)		4 items	11.80	.710	4 items	12.02	.703	4 items	11.89	.709
1	I’ve often had trouble making sense of the things I have to study	.744			.726			.736		
3	Much of what I’ve learned seems no more than lots of unrelated bits & pieces in my mind	.556			.605			.588		
7	Topics are presented in such complicated ways that I often can’t see what they mean	.622			.628			.630		
9	Often I have to study over and over things that don’t really make much sense to me	.546			.480			.509		

**Table 4:** Comparison between 2yCCT students and freshmen entrants on the factor analysis in the subscale of generic skills and development.

Generic Skills and Development (7 items)		2yCCT Students n = 840, KMO = .913			Freshmen Entrants n = 978, KMO = .888			Overall N = 1817, KMO = .904		
Questions		Factor Loading	Variance 54.70	α	Factor Loading	Variance 49.50	α	Factor Loading	Variance 52.07	α
		7 items	54.70	.891	7 items	49.50	.866	7 items	52.07	.879
1	I have learnt to apply theoretical knowledge to practice	.622			.528			.573		
2	I have learnt to develop cooperation and interpersonal skills (LEARN: “My studies have developed my cooperation and interaction skills”)	.699			.689			.694		
3	I have learnt to analyze and categorize information	.772			.751			.760		
4	I have learnt to see things from different points of view	.790			.756			.773		
5	I have learnt to look at things critically	.774			.767			.771		
6	I have learnt to make arguments and look for different solutions	.793			.756			.776		
7	I have learnt to develop new ideas	.711			.644			.680		

**Table 5:** Comparison between 2yCCT students and freshmen entrants on the factor analysis in the subscales of Self-efficacy and study workload & stress.

Self-efficacy (5 items)		2yCCT Students n = 841, KMO = .864			Freshmen Entrants n = 978, KMO = .852			Overall n = 1819, KMO = .860		
Questions		Factor Loading	Variance 57.22	α	Factor Loading	Variance 54.02	α	Factorm Loading	Variance 55.70	α
		5 items	57.22	.868	5 items	54.02	.851	5 items	55.70	.861
1	I believe I will do well in my studies	.792			.807			.799		
2	I’m certain I can understand the most difficult material in my studies	.718			.693			.706		
3	I’m confident I can understand the basic concepts of my own study field	.729			.735			.732		
4	I expect to do well in my studies	.754			.679			.719		
5	I’m certain I can learn well the skills required in my study field	.787			.754			.772		
Study Workload and Stress (3 items)		2yCCT Students n = 841, KMO = .701			Freshmen Entrants n = 978, KMO = .695			Overall n = 1819, KMO = .700		
Questions		Factor Loading	Variance 55.10	α	Factor Loading	Variance 52.67	α	Factor Loading	Variance 54.38	α
		3 items	55.10	.785	3 items	52.67	.768	3 items	54.38	.780
1	The workload of my studies is too heavy & causes too much study-related stress	.758			.723			.742		
2	I put too much effort into my studies	.688			.679			.689		
3	I am suffering from a high level of study-related stress	.778			.772			.779		

The reliabilities of a total of eight factors were calculated using Cronbach alphas and were found to be acceptable (Table 6). Using “studies workload stress” as an independent variable, a collinearity diagnostic test was conducted. The tolerance values ranged from 0.323 to 0.896, and the VIF values ranged from 1.116 to 3.097. Since the VIF values were between 1 and 10, we concluded that no instance of excessive collinearity among the independent variables was evident in the data.

**Table 6:** The construction of the factors and their reliabilities.

	Cronbach’s Alpha
Teaching and Learning Environment:	
Teaching for understanding & encouraging learning (11 items)	.918
Peer support (3 items)	.771
Alignment & constructive feedback (7 items)	.889
Approaches to Learning and Studying:	
Deep and organized approach (8 items)	.837
Surface approach (4 items)	.709
Self-efficacy (5 items)	.861
Study Workload Stress (3 items)	.780
Generic Skills (7 items)	.879

From the results of CFA on approaches to learning labelled “deep and organized approach”, and “surface approach”, the chi-square test (χ^2^ = 819.828, *df* = 53, *p* < 0.0001) indicated a poor fit; however, this was expected due to the current large sample. The fit indices (GFI = 0.927, CFI = 0.874, TLI = 0.843, RMSEA = 0.089) were borderline. Inspection of the modification indices suggested that low covariances between the error terms for items 5 and 6, items 8 and 10, as well as items 11 and 12 could significantly improve the model fit, resulting in improved indices (GFI = 0.961, CFI = 0.935, TLI = 0.914, RMSEA = 0.066).

For the CFA on students’ perceptions of the teaching and learning environment with three scales: “Interest, relevance and staff support”, “Peer support”, and “Constructive alignment and feedback”; again the chi-square test indicated a poor fit (χ^2^ = 1520.479, *df* = 186, *p* < 0.0001), while other indices showed quite a good fit of the model (GFI = 0.923, CFI = 0.937, TLI = 0.929, RMSEA = 0.063). Based on the analyses, we concluded that the factor structures of the subscales were acceptable. Higher order factor analysis was performed using the Schmid-Leiman solution [84]. Only one second-order factor was extracted from the first-order factor correlation matrix which is in line with the previous study using TLE scales. Together they measure the so called academic quality, and therefore, correlate highly to each other [68].

### 3.3. Differences between two groups of students on the study variables

Since the results of factor analyses between the two groups of students were highly similar, the factor structure that emerged from the whole data was used for further analysis. Table 7 shows the average scores of the students. It was found that the freshmen entrants scored statistically significantly higher on the perceptions of teaching for understanding & encouraging learning, peer support, and self-efficacy beliefs. Although both groups scored relatively low on the surface approach, the 2yCCT students scored statistically significantly higher (p < 0.0001) than the freshmen entrants in this respect. Students’ perceived study workload stress was also significantly higher for the 2yCCT students. There were no statistically significant differences in students’ perceptions of alignment & constructive feedback, or deep & organized approach to learning.

**Table 7:** Comparisons between 2yCCT students and freshmen entrants in eight factors.

Factors	2yCCT Students	Freshmen Entrants	Overall	p-value for T-test	t-value	degrees of freedom
	Mean (SD)	Mean (SD)	Mean (SD)			
Teaching for understanding & encouraging learning	3.58 (0.63)	3.64 (0.54)	3.61 (0.58)	**.038**	2.074	1672
Peer support	3.75 (0.66)	3.83 (0.62)	3.79 (0.64)	**.010**	2.586	1817
Alignment & constructive feedback	3.53 (0.63)	3.59 (0.60)	3.56 (0.62)	.056	1.914	1816
Deep & organized approach	3.54 (0.51)	3.54 (0.50)	3.54 (0.51)	.760	0.305	1817
Surface approach	3.24 (0.61)	3.12 (0.62)	3.17 (0.62)	**<.001**	-3.984	1817
Self-efficacy	3.50 (0.68)	3.58 (0.61)	3.54 (0.65)	**.015**	2.445	1707
Generic skills	3.67 (0.59)	3.71 (0.52)	3.69 (0.55)	.080	1.749	1697
Study workload stress	3.45 (0.77)	3.23 (0.76)	3.33 (0.77)	**<.001**	-6.102	1817

To illuminate the students’ strengths, their responses to the individual items were examined. Comparisons at the item level revealed that the freshmen entrants had strengths across all the factors; while the 2yCCT students had weaknesses and difficulties in approaches to learning and studying, as well as higher study workload stress (Table 8). The comparison revealed strengths and areas of development in the freshmen entrants and gave useful information for student support and quality enhancement for the 2yCCT students.

**Table 8:** Strengths/Weaknesses of the 2yCCT students and freshmen entrants (items scored statistically significant).

Strengths of freshmen entrants (scores significantly higher than those of 2yCCT students):
It is clear to me what I am expected to learn in subjects (Teaching and learning environment, Item 1)* t:2.167 *df*: 1731
What we are taught seems to match what we are supposed to learn (Teaching and learning environment, Item 3)** t: 3.104 *df*: 1704
Students support each other and try to give help when it is needed (Teaching and learning environment, Item 8)** t: 2.953 *df*: 1717
I can generally work comfortably with other students (Teaching and learning environment, Item 15)* t: 2.303 *df*: 1734
It is clear to me what is expected in the assessed work (Teaching and learning environment, Item 18)** t: 2.738 *df*: 1735
I can see how the subject assessment fits in with what I am supposed to learn (Teaching and learning environment, Item 19)** t: 2.892 *df*: 1716
The subject assessment helps me to make connections to my existing knowledge or experience (Teaching and learning environment, Item 21)* t: 2.026 *df*: 1728
I believe I will do well in my studies (Self-efficacy, Item 1)* t: 2.002 *df*: 1817
I’m certain I can understand the most difficult material in my studies (Self-efficacy, Item 2)* t: 2.301 *df*: 1817
I have learnt to make arguments and look for different solutions (Generic skills and development, Item 6)* t: 2.229 *df*: 1733
I have learnt to develop new ideas (Generic skills and development, Item 7)* t: 2.054 *df*: 1718
Weaknesses/difficulties of 2yCCT students (scores significantly higher than those of freshmen entrants):
I’ve often had trouble making sense of the things I have to study (Approaches to learning and studying, Item 1)*** t: -3.772 *df*: 1817
Much of what I’ve learned seems no more than lots of unrelated bits & pieces in my mind (Approaches to learning and studying, Item 3)** t: -2.753 *df*: 1817
Topics are presented in such complicated ways that I often can’t see what they mean (Approaches to learning and studying, Item 7)* t: -2.570 *df*: 1817
Often I have to study over and over things that don’t really make much sense to me (Approaches to learning and studying, Item 9)* t: -2.521 *df*: 1817
The workload of my studies is too heavy & causes too much study-related stress (Study workload and stress, Item 1)*** t: -4.249 *df*: 1817
I put too much effort into my studies (Study workload and stress, Item 2)*** t: -5.529 *df*: 1752
I am suffering from a high level of study-related stress (Study workload and stress, Item 3)*** t: -5.390 *df*: 1817
------------------------

*p < 0.05,

**p<0.01,

***p<0.001.

t: t-value; *df*: degrees of freedom.

### 3.4. Correlations among study variables and study workload stress in the two groups of students

In both groups, the study workload stress was positively and significantly correlated to deep & organized studying, and surface approach. However, it was negatively and significantly correlated to teaching for understanding & encouraging learning. It was also correlated negatively to alignment & constructive feedback in the 2yCCT students, but this correlation was not significant in the freshmen entrants (Table 9).

**Table 9:** Correlations between study variables and study workload stress.

**2yCCT students**								
Factors	1	2	3	4	5	6	7	8
1. Study workload stress	1							
2. Teaching for understanding & encouraging learning	–0.074*	1						
3. Peer support	–0.030	0.627**	1					
4. Alignment & constructive feedback	–0.112**	0.793**	0.636**	1				
5. Deep & organized approach	0.168**	0.481**	0.441**	0.514**	1			
6. Surface approach	0.293**	–0.103**	0.003	–0.030	0.212**	1		
7. Self-efficacy	0.017	0.558**	0.403**	0.526**	0.582**	–0.059	1	
8. Generic skills	0.036	0.584**	0.527**	0.570**	0.513**	–0.046	0.600**	1
**Freshmen Entrants**								
Factors	1	2	3	4	5	6	7	8
1. Study workload stress	1							
2. Teaching for understanding & encouraging learning	–0.026	1						
3. Peer support	–0.048	0.607**	1					
4. Alignment & constructive feedback	–0.043	0.789**	0.589**	1				
5. Deep & organized approach	0.185**	0.506**	0.435**	0.525**	1			
6. Surface approach	0.359**	–0.010	0.005	0.039	0.206**	1		
7. Self-efficacy	0.010	0.478**	0.369**	0.479**	0.612**	–0.023	1	
8. Generic skills	0.075*	0.585**	0.480**	0.570**	0.566**	–0.003	0.496**	1
**Overall**													
Factors	1	2	3	4	5	6	7	8
1. Study workload stress	1							
2. Teaching for understanding & encouraging learning	–0.057*	1						
3. Peer support	–0.047*	0.617**	1					
4. Alignment & constructive feedback	–0.081**	0.791**	0.613**	1				
5. Deep & organized studying	0.174**	0.492**	0.437**	0.519**	1			
6. Surface approach	0.337**	–0.060*	–0.002	0.002	0.207**	1		
7. Self-efficacy	0.005	0.521**	0.388**	0.504**	0.596**	–0.045	1	
8. Generic skills	0.049*	0.585**	0.504**	0.571**	0.539**	–0.027	0.551**	1

*p < 0.05,

**p<0.01

Furthermore, in general, the subscales of the teaching and learning environment were positively and significantly related to deep & organized approach, self-efficacy, and generic skills, but negatively or not significantly related to surface approach. We can also observe that deep & organized approach was positive significantly related to surface approach, self-efficacy, and generic skills. On the other hand, surface approach was negatively, though not significantly, related to self-efficacy, and generic skills.

### 3.5. Factors affecting study workload stress

Table 10 shows that the surface approach to learning (β = 0.302, p < 0.001); deep & organized studying (β = 0.182, p < 0.001); alignment & constructive feedback (β = −0.227, p < 0.001); and generic skills (β = 0.089, p = 0.002) [F(4,1811) = 84.040, p < 0.001] were the predictors of study workload stress in the overall student data. In both groups, the surface approach to learning was the strongest predictor of study workload stress. Age and gender were adjusted because they could be predicted perfectly from one or more of the other independent variables.

**Table 10:** Factors affecting study workload and stress.

Regression	2yCCT Students	Freshmen Entrants	Overall
F(df), p	F(4,833) = 33.574, p<0.001	F(5,972) = 39.791, p<0.001	F(4,1811) = 84.040, p<0.001
R, R^2^	R = 0.373, R^2^ = 0.139	R = 0.412, R^2^ = 0.170	R = 0.396, R^2^ = 0.157
Related factor(s)	Surface approach	Surface approach	Surface approach
	(β = 0.246, p<0.001)	(β = 0.330, p<0.001)	(β = 0.302, p<0.001)
	Deep & organized studying	Deep & organized studying	Deep & organized studying
	(β = 0.203, p<0.001)	(β = 0.177, p<0.001)	(β = 0.182, p<0.001)
	Alignment & constructive feedback	Alignment & constructive feedback	Alignment & constructive feedback
	(β = –0.261, p<0.001)	(β = –0.160, p<0.001)	(β = –0.227, p<0.001)
	Generic skills	Generic skills	Generic skills
	(β = 0.093, p = 0.025)	(β = 0.106, p = 0.007)	(β = 0.089, p = 0.002)
		Peer support	
		(β = –0.083, p = 0.027)	

adjusted for age and gender

## 4. Discussion

Our study has probably been the first to adopt the well-validated HowULearn questionnaire from the European to an Asian higher education context to assess the differences between CCT students and freshmen entrants with regard to their learning environment and study workload stress. Our findings indicated that the HowULearn questionnaire was a relatively robust cross-cultural instrument [Hypothesis 1 was supported]. In general, the results suggested that the CCT students had less positive learning experiences and higher study workload stress than the freshmen entrants [Hypothesis 2 was supported]. The factors affecting study workload stress were comparable for both groups of students, namely approaches to learning, alignment & constructive feedback, generic skills, and peer support [Hypothesis 3 was not supported].

### 4.1. HowULearn questionnaire in Asian higher education context

Two of the five HowULearn questionnaire scales, teaching and learning environment and approaches to learning, have been well validated, originally in Finland [46,65] and also in other contexts, Finland, England [69], Greece [85]; and Denmark [66]. Our study found the items in all five scales were relevant, with factor loading variances greater than 50% and Cronbach alphas greater than 0.70. In addition, convergent validity was supported by the positive relationship between experiences of the teaching and learning environment and the deep approaches to learning, as well as the negative relationship with surface approaches to learning [66,85,86].

Our study also found that the deep and organised approach to learning was associated positively with generic skills, while the surface approach was associated negatively. The factors loaded in our study were comparable with those validated in European higher education except for the case of two scales—only three factors were loaded in our study for teaching and learning environment and two factors for student approach to learning. For teaching and learning environment, the factor loadings were found to be inconsistent with previous studies. Although six factors (i.e., interest and relevance, peer support, staff enthusiasm and support, teaching for understanding, alignment and constructive feedback) had been identified previously in Finland [46,65] and Denmark [66], four factors (i.e., congruence & coherence in course organization, teaching for understanding & encouraging learning, support from other students, and integrative learning & critical thinking) were identified in Greek university students [86]. A review of the items loaded in these studies indicated that the three factors loaded in “Peer support” were consistent across European and Asian university students. Our 11-item factor of “Teaching for understanding & encouraging learning” was comparable to the Greek 10-item factor [86]. Furthermore, our 7-item “Alignment & constructive feedback” was comparable to the combination of the 2 factors of “Alignment” and “Constructive feedback” in Finnish university students [46,65]. This may be related to the well-known fact that the education systems in eastern countries are more competitive and teacher-centered, while those in western countries tend to be more flexible and student-centered [87]. Comparisons of eastern and western teachers have suggested that the eastern teachers focus more on content but the western ones place more emphasis on students when developing their lesson plans [42]. Thus, the perceived teaching and learning environments might differ across countries.

In terms of learning approaches, the 4-items for surface learning approaches were consistent across European and Asian university students, indicating that a dimension of unreflected studying, consisting of difficulty seeing the bigger picture and focusing on memorisation, can be found in both contexts. However, we could only identify one 8-item factor of “deep approach & organized studying”, probably because of their inter-connected relationship. The deep approach has been used to describe students who can apply critical thinking and organization skills to create their own understanding of their study [38]. In other words, a deep approach might include some elements of organizational skills. This is also in line with many studies reporting that deep approaches to learning are usually correlated positively with organised study [36]. However, more recent studies conducted in European contexts have also shown that, at the individual level, the student may use a combination of deep approach and unorganised studying. In such a combination, students are highly motivated and interested in their studying but are not able to manage their time and effort [46]. This raises an interesting question, whether such a combination is applicable in the Asian context. Thus, the results highlight that more cross-cultural studies are required to further validate the HowULearn instrument, particularly the constructs of teaching & learning environment and approaches to learning.

### 4.2. Differences between CCT students and freshmen entrants

#### 4.2.1. Teaching and learning environment

Our study results showed that the mean values of the three subscales (i.e., teaching for understanding & encouraging learning, peer support and alignment & constructive feedback) were all above 3.5 (out of five). These results reflect that the students generally perceived their teaching and learning environments positively. However, the CCT students perceived their environments to be significantly lower than the freshmen entrants did in terms of “teaching for understanding & encouraging learning” (p = 0.038) and “peer support” (p = 0.01).

Although CCT students come from associate degree or higher diploma programmes in relevant disciplines, their knowledge backgrounds can be diverse. While they are required to study for two years in university, some of them might not have sufficient discipline knowledge to begin their study. These students might perceive that the teaching and learning environment does not facilitate their understanding. On the contrary, those who have substantial discipline knowledge might lose interest in studying. This could be intensified when they are required to study some subjects which they have studied partly before. They might overlook the depths of the parts of the subjects which they have not studied yet in their associate degree or higher diploma programmes. These mismatched expectations may account for their losing interest and their perceptions of the programme relevance [29], thus affecting their experiences of teaching for understanding and encouraging their learning. Also, the transfer shock and the campus culture shock discussed in the literature are factors possibly leading to this difference between CCT students and freshmen entrants. In terms of support, our findings are understandable because these CCT students spend only two years in their programmes. Their bonding with staff and peers is supposed to be weaker than that of the freshmen entrants. Nevertheless, the results of this study showed no significant differences in “alignment & constructive feedback” (p = 0.056). As the students might not be identified specifically in their university courses as CCT students or freshmen entrants, they have equal opportunities to receive feedback from different parties.

#### 4.2.2. Approaches to learning

Webster and Yang [88] suggested that a facilitated teaching and learning environment would develop deep and organised approaches toward learning. Our study also revealed this pattern. In theory, a deep approach emphasises understanding the meaning and an integration of interesting knowledge, whereas organized studying focuses on study skills, such as time management and the use of a systematic learning approach in an effective way so as to achieve high academic results [89]. A skilful student who is able to apply deep approaches to learning falls into this category. In fact, Chinese learners are considered as strategic learners [90]. Our study results demonstrated that both the CCT students and the freshmen entrants adopted deep and organised approaches to learning (p = 0.76). This echoes the finding of Sakurai and colleagues [41], that Chinese students use deep and organised studying approaches is much the same ways their international counterparts do. As the students in both groups favour deep approaches to learning, the CCT students scored significantly higher than the freshmen entrants on surface approaches (p < 0.0001). Our in-depth analyses of the items on which there were statistically significant differences between the two groups of students (Table 8) illustrated that the CCT students generally had heavy study workloads and encountered issues concerned with linking unrelated bits together, and understanding complicated topics. In short, they faced a lot of sense-making issues, with limited time to comprehend the learning process. These might explain why the CCT students were reported as having higher attrition rates and lower academic performances than the freshmen entrants in general [4]. Although they tended to adopt deep and organised learning approaches throughout their two years of study, a surface approach seemed to provide a short-term solution for them. This finding is similar to that of Fryer and Vermunt [43], who argued that Chinese and Japanese students are able to apply both surface and deep approaches in learning. However, our study revealed that the CCT students used surface approaches in learning more than their freshmen entrant counterparts.

#### 4.2.3. Self-efficacy and generic skills

Our study results found that both the CCT students and the freshmen entrants perceived their self-efficacy and generic skills to be positive, with mean values of 3.5 or above (out of five). That is, the students in general agreed that they were confident of doing well in their studies. Although there were no differences in the scores of the CCT students and freshmen entrants for “generic skills”, a significant difference was found in their “self-efficacy beliefs” (p = 0.015). The CCT students were less confident than their freshmen entrant peers. As self-efficacy is associated with academic performance [70], we believe that some CCT students might not perform as well as freshmen entrants in the same teaching and learning environment, because they lack confidence. In most eastern countries, students’ status is evaluated based on their acceptance in the university [91, 92]. Only students who cannot gain admittance to university will go to community colleges. They might be considered as failures if they cannot get into university [91,92]. This culture certainly affects the students’ confidence in their studies. Herrera and colleagues [88] advocated the transfer receptive culture, i.e., the changes in the CCT students’ self-perceptions from “failure” to “success”. Strategies should be implemented to change the university culture to one that encourages students to believe they will be successful because they are CCT students, instead of despite being CCT students. In addition, universities should have the responsibility and commitment to provide adequate support to ensure CCT students transfer successfully [93]. Further studies should be carried out to investigate what is needed to facilitate this successful transfer, particularly in Asian contexts.

#### 4.2.4. Study workload stress and its predictors

Our study results suggested that the students had experienced certain levels of perceived workload stress. The CCT students perceived their workload intensity to be higher than the freshmen entrants did theirs; the difference was significant (p < 0.0001). As discussed, the CCT students had lower self-efficacy, less peer support, heavier study workloads, encountered more sense-making issues, put more effort into their studies, and were more likely to adopt surface approaches to their learning. These difficulties induced higher perceived study workload stress. This finding is consistent with Cheung [12]. Our further regression analyses identified the predictors affecting students’ study workload stress, namely “surface approach” (β = 0.302, p < 0.001), “deep and organized studying” (β = 0.182, p < 0.001), “alignment & constructive feedback” (β = -0.227, p < 0.001) and “generic skills” (β = 0.089, p = 0.002).

Kember and Leung [94] and Kember [54] have stated that the impact of perceived workload and the use of a surface approach is bi-directional. That is, a heavier workload motivates students to adopt surface approaches as a shortcut. By memorizing without thorough understanding, their perceived or actual workloads will become heavier [54,94]. However, our results provided a slightly different viewpoint. The CCT student group indicated more use of surface approaches than the freshmen entrants did, but in this group there was a lower correlation between “surface approach” and “study workload stress” (β = 0.246, p < 0.001 for the CCT students, while β = 0.330, p < 0.001 for the freshmen entrants). This implies that, when a student who does not adopt surface approaches very frequently does so, s/he is more sensitive to an increase in perceived workload stress. This is possible because, when a learner is accustomed to a particular study approach, a change of the approach can cause resistance and adaptation issues.

While Hernesniemi and colleagues [95] reported no evidence to associate the adoption of deep approaches with perceptions of the workload, our study of Chinese students suggested that a deep and organised approach was one factor leading to perceived workload stress. One possible explanation is the inclusion of the organised studying element. To recall, organised studying is related to learning strategy. Students need to devote additional time and effort to managing their study effectively so as to attain high academic achievement. The additional input and result-oriented mindset could result in workload and stress issues.

The relationship between “alignment & constructive feedback” and “study workload stress” was found to be negatively correlated (β = –0.227, p < 0.001). This finding is intuitive because if continuous feedback is given to students, they can evaluate their performances in a timely way. It also opens an opportunity for communication, encouragement and improvement so as to mitigate stress. Breaking down a big assessment component into small pieces is also helpful in spreading the workload. One aspect which cannot be overlooked is “generic skills”, as this has also been found to be a predictor of study workload stress. One possible explanation is that generic skills such as critical thinking, analytical skills and practical skills cannot be taught directly. They have to be learned and experienced. In fact, they facilitate a deep and organized studying approach. If the students cannot master the skills well, they tend to adopt surface approaches and to suffer from the workload stress issues [54,94].

The only difference between the CCT students and the freshmen entrants was “peer support”, which was found to be a predictor of study workload stress for the freshmen entrants only. The relationship is negative (β = –0.083, p = 0.027). Two points emerge here. First, this relationship is consistent with Kember’s [54] statement that peer support could alleviate perceived workload stress, particularly in Chinese students who are used to studying together with their peers. Second, the freshmen entrants obtained more support from their peers as they spent twice the time spent by the CCT students on peer-support activities. This explains why this predictor only had a significant effect on study workload and stress level for the freshmen entrants.

### 4.3. Implications

Our study results reflect the challenges faced by the CCT students as compared with freshmen entrants, as they strive for the same academic achievement (i.e., a bachelor’s degree). Of the predictors identified, approaches to learning (either surface or deep & organizing studying) and generic skills require students to invest additional time and effort in managing their study, thus increasing their study workloads and stress. For CCT students, time is a pressing issue. In western countries, most CCT students are non-traditional students (i.e., older university students) with family, work and study responsibilities [96,97]. In Asian countries, such as Hong Kong, the ages of CCT students and freshmen entrants are similar. This is because the education systems in Asian countries encourage students to continue their degree studies directly after secondary school education. However, CCT students have only two years while freshmen entrants have four years to complete their university studies. Thus, CCT students in western and eastern contexts share a common challenge—the time factor. In order to enhance CCT students’ learning experiences, it is of paramount importance to increase the articulation between community colleges and university degree programmes. Previous studies have found that students with more credit transfers have better university learning experiences [28,98]. An effective transfer will lead to improved academic performances, but failure may lead to students dropping out of programmes [99]. Generally, university programmes with similar natures compete with one another to recruit potential students. This competition hinders the development of common credit transfer patterns for students coming from similar disciplines. As a result, each university programme might consider graduates from a particular programme and from a particular community college. There is a need to have a paradigm shift from competition to collaboration among different stakeholders (such as community colleges, universities, and policy makers in the government) to facilitate the transparency of the subject alignment for credit transfers from community college to degree programmes. In addition, the “transfer receptive culture” that changes the perceptions of CCT students from “failure” to “success” [93] is essential. This positive collaborative culture will further enhance the review of the alignment, with the ultimate goal of improving the number of subjects considered for credit transfers, as well as the learning experience. With the reduction of the study workload, the associated stress would also be reduced correspondingly. Flexibility or extensions of the study period, instead of maintaining a rigid two-year study, might also be a way to reduce the study workload and stress. With these strategies implemented, CCT students will have more curricular and mental space for their personal and professional development.

### 4.4. Limitations

The involvement of just one university in this study limits its generalizability to other universities. However, the generalizability of the study results to the university studied might be high because of the large sample size, with the involvement of all faculties of the university. On the other hand, the retrospective nature of the cross-sectional study design has its weaknesses, such as recall bias.

## 5. Conclusion

This study clearly shows that the two students groups differ in how they experience their teaching-learning environment, in their approaches to learning, self-efficacy and study workload stress. This indicates that the two groups should be approached differently. The CCT students might need more encouragement in their learning and help with relating course content in order to see the bigger picture of what they are studying. This might help to reduce their perceived study workload stress. The study also highlights that measuring the dimensions of students’ experiences of the teaching-learning environment, their learning processes and study workloads, can give crucial information for educational institutions for developing their teaching across different cultures.

## Supporting information

S1 Data (PDF)
