# Supplementary material for: Differences in study workload stress and its associated factors between transfer students and freshmen entrants in an Asian higher education context
Source: PLoS One. 2020 May 15;15(5):e0233022. doi: 10.1371/journal.pone.0233022 (PMC7228073; doi:10.1371/journal.pone.0233022)
Supplement: S1 Data — (PDF) [file pone.0233022.s001.pdf]

Date ____________________

Questionnaire to Assess Senior Year Admitted (SYA) Students' Needs

The questions and statements in the questionnaire are based on prior research on academic teaching and learning. There are no right or wrong answers. Please choose the most appropriate response that best fits your immediate reaction to each question. Do not spend a long time on each item. Your First Reaction is probably the best answer. Do not worry about projecting a good image. Your answers are CONFIDENTIAL.

Please write your answers in the space provided or blacken the appropriate bubble.

Are you a Senior Year Admitted (or Senior Year Intake) student? ① Yes ② No

Part 1. Background / Personal Information

1 Student ID#: ____________________

2 Programme of Study: ____________________

3 Name of Department/School: ____________________

4 Year of Birth (dd/mm/yyyy): ____________

5 Gender: ① Male ② Female

6 Year of Intake: ① 2014/15 ② 2015/16 ③ 2016/17 ④ 2017/18 ⑤ 2018/19

7 Please indicate your HKDSE results in the following subjects that you took:

			Grade
a	English Language		________
b	Chinese Language		________
c	Mathematics		________
d	Liberal Studies		________
e	Elective subject, please specify:	____________	________
f	Elective subject, please specify:	____________	________
g	Elective subject, please specify:	____________	________

If you did not take HKDSE, please provide the details of the public examination you have taken.

____

Part 2. General Development of Teaching

A Consider your studies as a whole at the University, please respond to the following questions and statements. Please show your level of agreement by blackening the bubble according to the scale of 1 to 5.

[1 = Completely disagree; 2 = Disagree; 3 = Neither disagree nor agree; 4 = Agree; 5 = Completely agree]

		1	2	3	4	5
1	It is clear to me what I am expected to learn in subjects	①	②	③	④	⑤
2	We are allowed some choices over what aspects of the subject to concentrate on in subjects	①	②	③	④	⑤
3	What we are taught seems to match what we are supposed to learn	①	②	③	④	⑤
4	I can see the relevance of most of what we are taught	①	②	③	④	⑤
5	Subjects have given me a sense of what goes on "behind the scenes" in the subject area	①	②	③	④	⑤
6	The teaching helps me to think about the evidence underpinning different views	①	②	③	④	⑤
7	Teaching encourages me to relate what I learned to issues in a wider context	①	②	③	④	⑤
8	Students support each other and try to give help when it is needed	①	②	③	④	⑤
9	I found most of what I learned in subjects really interesting	①	②	③	④	⑤
10	Academic staff try to share their enthusiasm about the subject with us	①	②	③	④	⑤
11	Talking with other students helps me to develop my understanding	①	②	③	④	⑤
12	Academic staff are patient in explaining things which seem difficult to grasp	①	②	③	④	⑤
13	I enjoyed participating in subjects	①	②	③	④	⑤
14	Academic staff help us to see how we are supposed to think and reach conclusions in subjects	①	②	③	④	⑤
15	I can generally work comfortably with other students	①	②	③	④	⑤
16	Subjects provide plenty of opportunities for me to discuss important ideas and topics	①	②	③	④	⑤
17	I receive enough feedback about my learning (e.g. assignment work)	①	②	③	④	⑤
18	It is clear to me what is expected in the assessed work (e.g. final examination)	①	②	③	④	⑤
19	I can see how the subject assessment fits in with what I am supposed to learn	①	②	③	④	⑤
20	The feedback given on my work helps me to improve my ways of learning & studying	①	②	③	④	⑤
21	The subject assessment helps me to make connections to my existing knowledge or experience	①	②	③	④	⑤
22	The feedback given on my subject assessments helps to clarify things I hadn't fully understood	①	②	③	④	⑤

B Studying and Learning

Consider your studies as a whole at the University and respond to the following questions and statements. Please show your level of agreement by blackening the bubble according to the scale of 1 to 5.

[1 = Completely disagree; 2 = Disagree; 3 = Neither disagree nor agree; 4 = Agree; 5 = Completely agree]

		1	2	3	4	5
1	I've often had trouble making sense of the things I have to study	①	②	③	④	⑤
2	I put a lot of effort into my studying	①	②	③	④	⑤
3	Much of what I've learned seems no more than lots of unrelated bits & pieces in my mind	①	②	③	④	⑤
4	On the whole, I've been systematic and organized in my studying	①	②	③	④	⑤
5	Ideas I've come across in my academic reading set me off on long chains of thought	①	②	③	④	⑤
6	I look at evidence carefully to reach my own conclusion about what I'm studying	①	②	③	④	⑤
7	Topics are presented in such complicated ways that I often can't see what they mean	①	②	③	④	⑤
8	I organize my study time carefully to make the best use of it	①	②	③	④	⑤
9	Often I have to study over and over things that don't really make much sense to me	①	②	③	④	⑤
10	I carefully prioritise my time to make sure I can fit everything in	①	②	③	④	⑤
11	I try to relate new material, as I am reading it, to what I already know on that topic	①	②	③	④	⑤
12	I try to relate what I have learned in one subject to what I learn in other subjects	①	②	③	④	⑤

C Self-efficacy Beliefs

Consider your studies as a whole at the University, please respond to the following questions and statements. Please show your level of agreement by blackening the bubble according to the scale of 1 to 5.

[1 = Completely disagree; 2 = Disagree; 3 = Neither disagree nor agree; 4 = Agree; 5 = Completely agree]

		1	2	3	4	5
1	I believe I will do well in my studies	①	②	③	④	⑤
2	I'm certain I can understand the most difficult material in my studies	①	②	③	④	⑤
3	I'm confident I can understand the basic concepts of my own study field	①	②	③	④	⑤
4	I expect to do well in my studies	①	②	③	④	⑤
5	I'm certain I can learn well the skills required in my study field	①	②	③	④	⑤

D The Workload of Studies and Study-related Stress at the Univresity

Consider your studies as a whole at the University, please show your level of agreement to each of the following statement by blackening the bubble according to the scale of 1 to 5.

[1 = Completely disagree; 2 = Disagree; 3 = Neither disagree nor agree; 4 = Agree; 5 = Completely agree]

		1	2	3	4	5
1	The workload of my studies is too heavy & causes too much study-related stress	①	②	③	④	⑤
2	I put too much effort into my studies	①	②	③	④	⑤
3	I am suffering from a high level of study-related stress	①	②	③	④	⑤

E General Working Life Skills

Consider the development of your skills at the University studies, please show your level of agreement to each statement by blackening the bubble according to the scale of 1 to 5.

[1 = Completely disagree, 2 = Disagree, 3 = Neither disagree nor agree, 4 = Agree, 5 = Completely agree]

		1	2	3	4	5
1	I have learnt to apply theoretical knowledge to practice	①	②	③	④	⑤
2	I have learnt to develop cooperation and interpersonal skills	①	②	③	④	⑤
3	I have learnt to analyze and categorize information	①	②	③	④	⑤
4	I have learnt to see things from different points of view	①	②	③	④	⑤
5	I have learnt to look at things critically	①	②	③	④	⑤
6	I have learnt to make arguments and look for different solutions	①	②	③	④	⑤
7	I have learnt to develop new ideas	①	②	③	④	⑤

End of Questionnaire

Please check back to make sure that you have answered every question.

Thank you very much for spending time completing this questionnaire: it is much appreciated!
